# Supplementary material for: Id4 promotes the elimination of the pro-activation factor Ascl1 to maintain quiescence of adult hippocampal stem cells
Source: eLife. 2019 Sep 25;8:e48561. doi: 10.7554/eLife.48561 (PMC6805120; doi:10.7554/eLife.48561)
Supplement: Source data 1. [file elife-48561-data1.pdf]

Main Figures Summary Statistics

Figure 1D

Unpaired t test	
P value	0.017
P value summary	*
Significantly different (P < 0.05)?	Yes
One- or two-tailed P value?	Two-tailed
t, df	t=3.937 df=4
How big is the difference?	
Mean ± SEM of column A	83.81 ± 4.111, n=3
Mean ± SEM of column B	100 ± 0, n=3
Difference between means	16.19 ± 4.111
95% confidence interval	4.772 to 27.6
R squared (eta squared)	0.7949
F test to compare variances	
F, DFn, Dfd	Infinity, 2, 2
P value	<0.0001
P value summary	****
Significantly different (P < 0.05)?	Yes

Figure 1E

Unpaired t test	
P value	0.4294
P value summary	ns
Significantly different (P < 0.05)?	No
One- or two-tailed P value?	Two-tailed
t, df	t=0.7923 df=149
How big is the difference?	
Mean ± SEM of column G	92.78 ± 4.492, n=119
Mean ± SEM of column H	100 ± 5.407, n=32
Difference between means	7.223 ± 9.116
95% confidence interval	-10.79 to 25.24
R squared (eta squared)	0.004196
F test to compare variances	
F, DFn, Dfd	2.566, 118, 31
P value	0.0034
P value summary	**
Significantly different (P < 0.05)?	Yes

Figure 1G

dots/nucleus	
Unpaired t test	
P value	0.1018
P value summary	ns
Significantly different (P < 0.05)?	No
One- or two-tailed P value?	Two-tailed
t, df	t=1.653 df=91
How big is the difference?	
Mean ± SEM of column A	3.95 ± 0.8413, n=20
Mean ± SEM of column B	2.589 ± 0.3649, n=73
Difference between means	-1.361 ± 0.8234
95% confidence interval	-2.997 to 0.2747
R squared (eta squared)	0.02914
F test to compare variances	
F, DFn, Dfd	1.457, 19, 72
P value	0.2577
P value summary	ns
Significantly different (P < 0.05)?	No

Relative intensity/nucleus	
Unpaired t test	
P value	0.1128
P value summary	ns
Significantly different (P < 0.05)?	No
One- or two-tailed P value?	Two-tailed
t, df	t=1.601 df=91
How big is the difference?	
Mean ± SEM of column J	100 ± 8.859, n=20
Mean ± SEM of column K	86.51 ± 3.689, n=73
Difference between means	-13.49 ± 8.423
95% confidence interval	-30.22 to 3.243
R squared (eta squared)	0.02741
F test to compare variances	
F, DFn, Dfd	1.58, 19, 72
P value	0.1704
P value summary	ns
Significantly different (P < 0.05)?	No

Figure 2A

Unpaired t test	
P value	0.9086
P value summary	ns
Significantly different (P < 0.05)?	No
One- or two-tailed P value?	Two-tailed
t, df	t=0.1197 df=6
How big is the difference?	
Mean ± SEM of column A	1.003 ± 0.08642, n=4
Mean ± SEM of column C	1.022 ± 0.1254, n=4
Difference between means	0.01823 ± 0.1523
95% confidence interval	-0.3543 to 0.3908
R squared (eta squared)	0.002383
F test to compare variances	
F, DFn, Dfd	2.104, 3, 3
P value	0.5569
P value summary	ns
Significantly different (P < 0.05)?	No

Figure 2C

Unpaired t test	
P value	0.0587
P value summary	ns
Significantly different (P < 0.05)?	No
One- or two-tailed P value?	Two-tailed
t, df	t=1.899 df=244
How big is the difference?	
Mean ± SEM of column J	100 ± 15.11, n=133
Mean ± SEM of column K	65.42 ± 8.571, n=113
Difference between means	-34.58 ± 18.2
95% confidence interval	-70.43 to 1.28
R squared (eta squared)	0.01457
F test to compare variances	
F, DFn, Dfd	3.659, 132, 112
P value	<0.0001
P value summary	****
Significantly different (P < 0.05)?	Yes

Figure 2E

Unpaired t test	
P value	7.09E-08
P value summary	****
Significantly different (P < 0.05)?	Yes
One- or two-tailed P value?	Two-tailed
t, df	t=5.66 df=156
How big is the difference?	
Mean ± SEM of column A	69.92 ± 4.128, n=79
Mean ± SEM of column C	45.6 ± 1.197, n=79
Difference between means	-24.32 ± 4.298
95% confidence interval	-32.81 to -15.83
R squared (eta squared)	0.1703
F test to compare variances	
F, DFn, Dfd	11.9, 78, 78
P value	<0.0000000001
P value summary	****
Significantly different (P < 0.05)?	Yes

Figure 2F

Unpaired t test	
P value	0.0363
P value summary	*
Significantly different (P < 0.05)?	Yes
One- or two-tailed P value?	Two-tailed
t, df	t=2.685 df=6
How big is the difference?	
Mean ± SEM of column D	1 ± 0.101, n=4
Mean ± SEM of column E	0.4756 ± 0.1672, n=4
Difference between means	-0.5244 ± 0.1953
95% confidence interval	-1.002 to -0.04642
R squared (eta squared)	0.5457
F test to compare variances	
F, DFn, Dfd	2.739, 3, 3
P value	0.4299
P value summary	ns
Significantly different (P < 0.05)?	No

Figure 2G

	Discovery?	P value	Mean1	Mean2
0				
5	No	0.387555649	1.178	1.435
10	No	0.020728625	1.2	1.902
30	No	0.026595256	1.084	3.106
60	No	0.023656159	1.028	3.314
120	No	0.232781025	1.169	2.391

Difference	SE of difference	t ratio	df	q value
-0.257	0.2653	0.9687	4	0.391431206
-0.702	0.1894	3.706	4	0.044768682
-2.022	0.5899	3.428	4	0.044768682
-2.286	0.6427	3.557	4	0.044768682
-1.222	0.8697	1.405	4	0.293886044

Figure 3A

Column B	Id1 BMP
vs.	vs.
Column A	Id1 FGF
Unpaired t test	
P value	0.0322
P value summary	*
Significantly different (P < 0.05)?	Yes
One- or two-tailed P value?	Two-tailed
t, df	t=3.221 df=4
How big is the difference?	
Mean ± SEM of column A	1 ± 0.3057, n=3
Mean ± SEM of column B	13.73 ± 3.94, n=3
Difference between means	12.73 ± 3.952
95% confidence interval	1.759 to 23.7
R squared (eta squared)	0.7218
F test to compare variances	
F, DFn, Dfd	166.1, 2, 2
P value	0.012
P value summary	*
Significantly different (P < 0.05)?	Yes

Column D	Id2 BMP
vs.	vs.
Column C	Id2 FGF
Unpaired t test	
P value	0.0746
P value summary	ns
Significantly different (P < 0.05)?	No
One- or two-tailed P value?	Two-tailed
t, df	t=3.453 df=2
How big is the difference?	
Mean ± SEM of column C	1 ± 0.1197, n=2
Mean ± SEM of column D	8.156 ± 2.069, n=2
Difference between means	7.156 ± 2.073
95% confidence interval	-1.761 to 16.07
R squared (eta squared)	0.8563
F test to compare variances	
F, DFn, Dfd	
P value	
P value summary	
Significantly different (P < 0.05)?	

Column F	Id3 BMP
vs.	vs.
Column E	Id3 FGF
Unpaired t test	
P value	0.00072921
P value summary	***
Significantly different (P < 0.05)?	Yes
One- or two-tailed P value?	Two-tailed
t, df	t=9.348 df=4
How big is the difference?	
Mean ± SEM of column E	1 ± 0.1075, n=3
Mean ± SEM of column F	6.8 ± 0.6111, n=3
Difference between means	5.8 ± 0.6205
95% confidence interval	4.077 to 7.523
R squared (eta squared)	0.9562
F test to compare variances	
F, DFn, Dfd	32.29, 2, 2
P value	0.0601
P value summary	ns
Significantly different (P < 0.05)?	No

Column H	Id4 BMP
vs.	vs.
Column G	Id4 FGF
Unpaired t test	
P value	0.0018
P value summary	**
Significantly different (P < 0.05)?	Yes
One- or two-tailed P value?	Two-tailed
t, df	t=7.418 df=4
How big is the difference?	
Mean ± SEM of column G	1 ± 0.281, n=3
Mean ± SEM of column H	27.44 ± 3.553, n=3
Difference between means	26.44 ± 3.564
95% confidence interval	16.54 to 36.33
R squared (eta squared)	0.9322
F test to compare variances	
F, DFn, Dfd	159.9, 2, 2
P value	0.0124
P value summary	*
Significantly different (P < 0.05)?	Yes

Figure 3C

Unpaired t test	
P value	0.000753309
P value summary	***
Significantly different (P < 0.05)?	Yes
One- or two-tailed P value?	Two-tailed
t, df	t=6.288 df=6
How big is the difference?	
Mean ± SEM of column D	1 ± 0.5676, n=4
Mean ± SEM of column E	4.627 ± 0.1026, n=4
Difference between means	3.627 ± 0.5769
95% confidence interval	2.216 to 5.039
R squared (eta squared)	0.8682
F test to compare variances	
F, DFn, Dfd	30.59, 3, 3
P value	0.0189
P value summary	*
Significantly different (P < 0.05)?	Yes

Figure 3E

Unpaired t test	
P value	2.32E-11
P value summary	****
Significantly different (P < 0.05)?	Yes
One- or two-tailed P value?	Two-tailed
t, df	t=7.205 df=156
How big is the difference?	
Mean ± SEM of column E	31.38 ± 1.249, n=79
Mean ± SEM of column F	48.98 ± 2.099, n=79
Difference between means	17.6 ± 2.443
95% confidence interval	12.78 to 22.43
R squared (eta squared)	0.2497
F test to compare variances	
F, DFn, Dfd	2.823, 78, 78
P value	<0.0001
P value summary	****
Significantly different (P < 0.05)?	Yes

Figure 3K

Unpaired t test	
P value	8.4E-11
P value summary	****
Significantly different (P < 0.05)?	Yes
One- or two-tailed P value?	Two-tailed
t, df	t=8.877 df=38
How big is the difference?	
Mean ± SEM of column A	105.7 ± 5.727, n=20
Mean ± SEM of column B	36.75 ± 5.25, n=20
Difference between means	-68.96 ± 7.769
95% confidence interval	-84.69 to -53.24
R squared (eta squared)	0.6746
F test to compare variances	
F, DFn, Dfd	1.19, 19, 19
P value	0.7084
P value summary	ns
Significantly different (P < 0.05)?	No

Figure 4D

Unpaired t test	
P value	3.05E-09
P value summary	****
Significantly different (P < 0.05)?	Yes
One- or two-tailed P value?	Two-tailed
t, df	t=6.233 df=184
How big is the difference?	
Mean ± SEM of column G	100 ± 6.793, n=93
Mean ± SEM of column H	54.1 ± 2.842, n=93
Difference between means	-45.9 ± 7.364
95% confidence interval	-60.42 to -31.37
R squared (eta squared)	0.1743
F test to compare variances	
F, DFn, Dfd	5.713, 92, 92
P value	<0.0001
P value summary	****
Significantly different (P < 0.05)?	Yes

Figure 4E

Paired t test	
P value	0.8739
P value summary	ns
Significantly different (P < 0.05)?	No
One- or two-tailed P value?	Two-tailed
t, df	t=0.1798 df=2
Number of pairs	3
How big is the difference?	
Mean of differences	-0.02549
SD of differences	0.2455
SEM of differences	0.1418
95% confidence interval	-0.6354 to 0.5844
R squared (partial eta squared)	0.01591
How effective was the pairing?	
Correlation coefficient (r)	-0.1451
P value (one tailed)	0.4537
P value summary	ns
Was the pairing significantly effective?	No

Figure 4G

Paired t test	
P value	0.05
P value summary	*
Significantly different (P < 0.05)?	Yes
One- or two-tailed P value?	Two-tailed
t, df	t=3.183 df=3
Number of pairs	4
How big is the difference?	
Mean of differences	-21.11
SD of differences	13.27
SEM of differences	6.633
95% confidence interval	-42.22 to -0.001007
R squared (partial eta squared)	0.7715
How effective was the pairing?	
Correlation coefficient (r)	-0.07008
P value (one tailed)	0.465
P value summary	ns
Was the pairing significantly effective?	No

Figure 4I

Paired t test	
P value	0.1148
P value summary	ns
Significantly different (P < 0.05)?	No
One- or two-tailed P value?	Two-tailed
t, df	t=2.691 df=2
Number of pairs	3
How big is the difference?	
Mean of differences	-8.833
SD of differences	5.686
SEM of differences	3.283
95% confidence interval	-22.96 to 5.292
R squared (partial eta squared)	0.7835
How effective was the pairing?	
Correlation coefficient (r)	0.7231
P value (one tailed)	0.2427
P value summary	ns
Was the pairing significantly effective?	No

Figure 4K

Unpaired t test	
P value	0.0131
P value summary	*
Significantly different (P < 0.05)?	Yes
One- or two-tailed P value?	Two-tailed
t, df	t=2.528 df=98
How big is the difference?	
Mean ± SEM of column G	100 ± 7.731, n=51
Mean ± SEM of column H	142.5 ± 15.16, n=49
Difference between means	42.52 ± 16.82
95% confidence interval	9.138 to 75.9
R squared (eta squared)	0.06121
F test to compare variances	
F, DFn, Dfd	3.694, 48, 50
P value	<0.0001
P value summary	****
Significantly different (P < 0.05)?	Yes

Figure 4L

Paired t test	
P value	0.0758
P value summary	ns
Significantly different (P < 0.05)?	No
One- or two-tailed P value?	Two-tailed
t, df	t=3.423 df=2
Number of pairs	3
How big is the difference?	
Mean of differences	-0.3785
SD of differences	0.1915
SEM of differences	0.1106
95% confidence interval	-0.8543 to 0.09724
R squared (partial eta squared)	0.8542
How effective was the pairing?	
Correlation coefficient (r)	0.746
P value (one tailed)	0.232
P value summary	ns
Was the pairing significantly effective?	No

Figure 4N

%KI67+	
Paired t test	
P value	0.0485
P value summary	*
Significantly different (P < 0.05)?	Yes
One- or two-tailed P value?	Two-tailed
t, df	t=4.372 df=2
Number of pairs	3
How big is the difference?	
Mean of differences	35.48
SD of differences	14.06
SEM of differences	8.116
95% confidence interval	0.5627 to 70.4

%EdU+	
Paired t test	
P value	0.085
P value summary	ns
Significantly different (P < 0.05)?	No
One- or two-tailed P value?	Two-tailed
t, df	t=3.208 df=2
Number of pairs	3
How big is the difference?	
Mean of differences	4.904
SD of differences	2.648
SEM of differences	1.529
95% confidence interval	-1.674 to 11.48

R squared (partial eta squared)	0.9053	R squared (partial eta squared)	0.8373
How effective was the pairing?		How effective was the pairing?	
Correlation coefficient (r)	0.9999	Correlation coefficient (r)	0.6835
P value (one tailed)	0.004	P value (one tailed)	0.2604
P value summary	**	P value summary	ns
Was the pairing significantly effective?	Yes	Was the pairing significantly effective?	No

Figure 5E

Gene name	logFC	logCPM	LR	PValue	FDR
Dll1	-2.16653603	5.151465748	44.15167485	3.04E-11	1.44E-09
Dll3	-1.605697967	0.774961332	22.23532548	2.41E-06	4.63E-05
Skp2	-2.263976591	5.234419896	73.61448822	9.50E-18	1.65E-15
Birc5	-2.351352295	5.058298298	50.3062338	1.32E-12	7.82E-11
Cdk1	-2.46413008	6.515691283	78.38606663	8.47E-19	2.09E-16
Cdk2	-1.016666564	5.861506896	12.71589534	0.000362561	0.00342061
Foxm1	-2.173282828	6.186910054	56.14989656	6.72E-14	5.20E-12
Egfr	-1.175714979	8.57757694	20.41016581	6.25E-06	0.000108167
Rrm2	-2.782875601	7.21832039	100.6004661	1.13E-23	1.28E-20
Fbl	-1.102638987	6.612927396	20.85305929	4.96E-06	8.75E-05

		Counts per Million	
Gene name	BLO567A1_FGF_GFP_1	BLO567A2_FGF_GFP_2	BLO567A3_FGF_GFP_3
Dll1	2063	1062	1002
Dll3	51	70	53
Skp2	1439	1722	1262
Birc5	1061	1480	1408
Cdk1	3037	3974	3997
Cdk2	1643	1959	1926
Foxm1	2307	3034	3130
Egfr	13198	11667	12829
Rrm2	5223	6983	6285
Fbl	3547	3093	2859

		Counts per Million	
Gene name	BLO567A10_FGF_ID4_1	BLO567A11_FGF_ID4_2	BLO567A12_FGF_ID4_3
Dll1	296	268	359
Dll3	21	17	18
Skp2	276	327	320
Birc5	200	183	402
Cdk1	610	552	847
Cdk2	734	723	1302
Foxm1	479	573	844
Egfr	6884	4936	4848
Rrm2	1067	756	864
Fbl	1497	1460	1472

Figure 6C

Number of families	1
Number of comparisons per family	3
Alpha	0.05

Tukey's multiple comparisons test	Mean Diff.	95.00% CI of diff.	Significant?	Summary	Adjusted P Value
Id4 fl/+ vs. Id4 fl/fl	-7.349	-14.2 to -0.4996	Yes	*	0.0381
Id4 fl/+ vs. Id4 +/+	3.504	-3.346 to 10.35	No	ns	0.3276
Id4 fl/fl vs. Id4 +/+	10.85	4.003 to 17.7	Yes	**	0.0067

Test details	Mean 1	Mean 2	Mean Diff.	SE of diff.	N1
Id4 fl/+ vs. Id4 fl/fl	7.937	15.29	-7.349	2.232	4
Id4 fl/+ vs. Id4 +/+	7.937	4.433	3.504	2.232	4
Id4 fl/fl vs. Id4 +/+	15.29	4.433	10.85	2.232	4

N2	q	DF
4	4.656	6
4	2.22	6
4	6.875	6

Figure 6D

Column R	Id4^{cKO}
vs.	vs.
Column Q	Control
Unpaired t test	
P value	2.01749E-05
P value summary	****
Significantly different (P < 0.05)?	Yes
One- or two-tailed P value?	Two-tailed
t, df	t=4.313 df=413
How big is the difference?	
Mean ± SEM of column Q	100 ± 6.474, n=236
Mean ± SEM of column R	161.6 ± 14, n=179
Difference between means	61.57 ± 14.27
95% confidence interval	33.51 to 89.63
R squared (eta squared)	0.0431
F test to compare variances	
F, DFn, Dfd	3.546, 178, 235
P value	<0.0001
P value summary	****
Significantly different (P < 0.05)?	Yes

Figure 6E

Number of families	1
Number of comparisons per family	3
Alpha	0.05

Tukey's multiple comparisons test	Mean Diff.	95.00% CI of diff.	Significant?	Summary	Adjusted P Value
Id4 +/fl vs. Id4 fl/fl	-2.069	-5.672 to 1.534	No	ns	0.3157
Id4 +/fl vs. Combined Id4wt	0.1065	-3.497 to 3.71	No	ns	0.9967
Id4 fl/fl vs. Combined Id4wt	2.176	-1.588 to 5.939	No	ns	0.3113

Test details	Mean 1	Mean 2	Mean Diff.	SE of diff.	n1
Id4 +/fl vs. Id4 fl/fl	3.151	5.22	-2.069	1.365	6
Id4 +/fl vs. Combined Id4wt	3.151	3.044	0.1065	1.365	6
Id4 fl/fl vs. Combined Id4wt	5.22	3.044	2.176	1.425	5

B-C
B-E
C-E

n2	q	DF

5	2.144	13
5	0.1103	13
5	2.158	13

Figure 6G

Number of families	1
Number of comparisons per family	3
Alpha	0.05

Tukey's multiple comparisons test	Mean Diff.	95.00% CI of diff.	Significant?	Summary	Adjusted P Value
Id4 +/+ vs. Id4 fl/+	-1.336	-7.003 to 4.331	No	ns	0.7595
Id4 +/+ vs. Id4 fl/fl	-9.593	-15.26 to -3.927	Yes	**	0.0049
Id4 fl/+ vs. Id4 fl/fl	-8.257	-13.92 to -2.591	Yes	*	0.0101

Test details	Mean 1	Mean 2	Mean Diff.	SE of diff.	N1
Id4 +/+ vs. Id4 fl/+	4.142	5.478	-1.336	1.847	4
Id4 +/+ vs. Id4 fl/fl	4.142	13.73	-9.593	1.847	4
Id4 fl/+ vs. Id4 fl/fl	5.478	13.73	-8.257	1.847	4

N2	q	DF
4	1.023	6
4	7.346	6
4	6.323	6

Figure 6H

Number of families	1
Number of comparisons per family	3
Alpha	0.05

Tukey's multiple comparisons test	Mean Diff.	95.00% CI of diff.	Significant?	Summary	Adjusted P Value
Id4 fl/+ vs. Id4 fl/fl	-2.53	-4.086 to -0.9735	Yes	**	0.0026
Id4 fl/+ vs. Combined Id4wt	-0.1114	-1.668 to 1.445	No	ns	0.9801
Id4 fl/fl vs. Combined Id4wt	2.419	0.8621 to 3.975	Yes	**	0.0036

Test details	Mean 1	Mean 2	Mean Diff.	SE of diff.	n1
Id4 fl/+ vs. Id4 fl/fl	2.658	5.188	-2.53	0.5834	5
Id4 fl/+ vs. Combined Id4wt	2.658	2.769	-0.1114	0.5834	5
Id4 fl/fl vs. Combined Id4wt	5.188	2.769	2.419	0.5834	5

C-D
C-E
D-E

n2	q	DF
5	6.133	12
5	0.2701	12
5	5.863	12

Figure 6J

Unpaired t test	
P value	0.0016
P value summary	**
Significantly different (P < 0.05)?	Yes
One- or two-tailed P value?	Two-tailed
t, df	t=7.658 df=4
How big is the difference?	
Mean ± SEM of column A	38.37 ± 4.561, n=3
Mean ± SEM of column B	74.16 ± 1.017, n=3
Difference between means	35.79 ± 4.673
95% confidence interval	22.81 to 48.76
R squared (eta squared)	0.9361
F test to compare variances	
F, DFn, Dfd	20.13, 2, 2
P value	0.0946
P value summary	ns
Significantly different (P < 0.05)?	No

Figure 6L

Unpaired t test	
P value	1.87108E-06
P value summary	****
Significantly different (P < 0.05)?	Yes
One- or two-tailed P value?	Two-tailed
t, df	t=42.28 df=4
How big is the difference?	
Mean ± SEM of column B	9.779 ± 1.049, n=3
Mean ± SEM of column D	75.31 ± 1.141, n=3
Difference between means	65.54 ± 1.55
95% confidence interval	61.23 to 69.84
R squared (eta squared)	0.9978
F test to compare variances	
F, DFn, Dfd	1.182, 2, 2
P value	0.9165
P value summary	ns
Significantly different (P < 0.05)?	No

Supplementary Figures Summary Statistics

Figure S1G

Multiple t tests

	Discovery?	P value	Mean1	Mean2	Difference	SE of difference	t ratio	df	q value
0									
5	No	0.387555649	1.178	1.435	-0.257	0.2653	0.9687	4	0.391431206
10	No	0.020728625	1.2	1.902	-0.702	0.1894	3.706	4	0.044768682
30	No	0.026595256	1.084	3.106	-2.022	0.5899	3.428	4	0.044768682
60	No	0.023656159	1.028	3.314	-2.286	0.6427	3.557	4	0.044768682
120	No	0.232781025	1.169	2.391	-1.222	0.8697	1.405	4	0.293886044

Figure S1I

Unpaired t test		
P value	<0.000000000000001	1E-15
P value summary	****	
Significantly different (P < 0.05)?	Yes	
One- or two-tailed P value?	Two-tailed	
t, df	t=15.66 df=415	
How big is the difference?		
Mean ± SEM of column M	34.62 ± 0.7523, n=218	
Mean ± SEM of column N	100 ± 4.292, n=199	
Difference between means	65.38 ± 4.175	
95% confidence interval	57.18 to 73.59	
R squared (eta squared)	0.3715	
F test to compare variances		
F, DFn, Dfd	29.71, 198, 217	
P value	<0.0001	
P value summary	****	
Significantly different (P < 0.05)?	Yes	

Figure S2L

Time	Discovery?	P value	Mean1	Mean2	Difference	SE of difference	t ratio	df	q value
0'	No	0.37390097	1	1	-1.667E-08	1.667E-08	1	4	0.18881999
15'	Yes	0.013268726	1.12	0.6859	0.4338	0.1023	4.24	4	0.008927355
30'	Yes	0.001575203	0.9927	0.4888	0.5039	0.06594	7.642	4	0.001590955
60'	Yes	0.000826463	0.9135	0.3477	0.5658	0.06253	9.049	4	0.001252091
120'	Yes	0.01473161	0.7754	0.1621	0.6133	0.1492	4.11	4	0.008927355
240'	Yes	0.000397588	0.3403	0.02546	0.3148	0.0288	10.93	4	0.001204693

Figure S3C

Unpaired t test	
P value	0.007
P value summary	**
Significantly different (P < 0.05)?	Yes
One- or two-tailed P value?	Two-tailed
t, df	t=2.86 df=36
How big is the difference?	
Mean ± SEM of column D	80.36 ± 7.817, n=19
Mean ± SEM of column E	114.2 ± 8.901, n=19
Difference between means	33.88 ± 11.85
95% confidence interval	9.857 to 57.91
R squared (eta squared)	0.1852
F test to compare variances	
F, DFn, Dfd	1.297, 18, 18
P value	0.5875
P value summary	ns
Significantly different (P < 0.05)?	No

Figure S3G

Unpaired t test	
P value	>0.9999
P value summary	ns
Significantly different (P < 0.05)?	No
One- or two-tailed P value?	Two-tailed
t, df	t=1.85e-009 df=373
How big is the difference?	
Mean ± SEM of column C	100 ± 4.972, n=57
Mean ± SEM of column D	100 ± 2.408, n=318
Difference between means	1.122e-008 ± 6.066
95% confidence interval	-11.93 to 11.93
R squared (eta squared)	9.173E-21
F test to compare variances	
F, DFn, Dfd	1.309, 317, 56
P value	0.2221
P value summary	ns
Significantly different (P < 0.05)?	No

Figure S4A

Unpaired t test	
P value	0.0196
P value summary	*
Significantly different (P < 0.05)?	Yes
One- or two-tailed P value?	Two-tailed
t, df	t=4.578 df=3
How big is the difference?	
Mean ± SEM of column B	6.912 ± 1.314, n=3
Mean ± SEM of column F	15.56 ± 1.111, n=2
Difference between means	8.644 ± 1.888
95% confidence interval	2.635 to 14.65
R squared (eta squared)	0.8748
F test to compare variances	
F, DFn, Dfd	
P value	
P value summary	
Significantly different (P < 0.05)?	

Figure S4B

Unpaired t test	
P value	0.4842
P value summary	ns
Significantly different (P < 0.05)?	No
One- or two-tailed P value?	Two-tailed
t, df	t=0.7701 df=4
How big is the difference?	
Mean ± SEM of column I	27.5 ± 5.204, n=3
Mean ± SEM of column L	22.61 ± 3.645, n=3
Difference between means	-4.893 ± 6.354
95% confidence interval	-22.53 to 12.75
R squared (eta squared)	0.1291
F test to compare variances	
F, DFn, Dfd	2.038, 2, 2
P value	0.6583
P value summary	ns
Significantly different (P < 0.05)?	No

Figure S4E

Column C	Id1	Column D	Id2	Column E	Id3	Column F	Id4
vs.	vs.	vs.	vs.	vs.	vs.	vs.	vs.
Column B	Empty	Column B	Empty	Column B	Empty	Column B	Empty
Unpaired t test		Unpaired t test		Unpaired t test		Unpaired t test	
P value	0.0022	P value	0.0034	P value	0.0051	P value	0.0006
P value summary	**	P value summary	**	P value summary	**	P value summary	***
Significantly different (P < 0.05)?	Yes	Significantly different (P < 0.05)?	Yes	Significantly different (P < 0.05)?	Yes	Significantly different (P < 0.05)?	Yes
One- or two-tailed P value?	Two-tailed	One- or two-tailed P value?	Two-tailed	One- or two-tailed P value?	Two-tailed	One- or two-tailed P value?	Two-tailed
t, df	t=6.984 df=4	t, df	t=6.217 df=4	t, df	t=5.573 df=4	t, df	t=9.791 df=4
How big is the difference?		How big is the difference?		How big is the difference?		How big is the difference?	
Mean ± SEM of column B	14.16 ± 0.9912, n=3	Mean ± SEM of column B	14.16 ± 0.9912, n=3	Mean ± SEM of column B	14.16 ± 0.9912, n=3	Mean ± SEM of column B	14.16 ± 0.9912, n=3
Mean ± SEM of column C	4.541 ± 0.9555, n=3	Mean ± SEM of column D	7.073 ± 0.5616, n=3	Mean ± SEM of column E	4.953 ± 1.321, n=3	Mean ± SEM of column F	2.889 ± 0.5845, n=3
Difference between means	-9.614 ± 1.377	Difference between means	-7.082 ± 1.139	Difference between means	-9.202 ± 1.651	Difference between means	-11.27 ± 1.151
95% confidence interval	-13.44 to -5.792	95% confidence interval	-10.24 to -3.919	95% confidence interval	-13.79 to -4.618	95% confidence interval	-14.46 to -8.072
R squared (eta squared)	0.9242	R squared (eta squared)	0.9062	R squared (eta squared)	0.8859	R squared (eta squared)	0.9599
F test to compare variances		F test to compare variances		F test to compare variances		F test to compare variances	
F, DFn, Dfd	1.076, 2, 2	F, DFn, Dfd	3.115, 2, 2	F, DFn, Dfd	1.775, 2, 2	F, DFn, Dfd	2.875, 2, 2
P value	0.9633	P value	0.486	P value	0.7207	P value	0.5161
P value summary	ns	P value summary	ns	P value summary	ns	P value summary	ns
Significantly different (P < 0.05)?	No	Significantly different (P < 0.05)?	No	Significantly different (P < 0.05)?	No	Significantly different (P < 0.05)?	No

Figure S4F

Column B	Id1 ABC 20nM each 48h	Column C	Id2 ABC 20nM each	Column D	Id3 ABC 20nM each	Column E	Id4 ABC 20nM each
vs.	vs.	vs.	vs.	vs.	vs.	vs.	vs.
Column A	Scrambled 60nM 48h	Column A	Scrambled 60nM 48h	Column A	Scrambled 60nM 48h	Column A	Scrambled 60nM 48h
Paired t test		Paired t test		Paired t test		Paired t test	
P value	0.4884	P value	0.0698	P value	0.6811	P value	0.6268
P value summary	ns	P value summary	ns	P value summary	ns	P value summary	ns
Significantly different (P < 0.05)?	No	Significantly different (P < 0.05)?	No	Significantly different (P < 0.05)?	No	Significantly different (P < 0.05)?	No
One- or two-tailed P value?	Two-tailed	One- or two-tailed P value?	Two-tailed	One- or two-tailed P value?	Two-tailed	One- or two-tailed P value?	Two-tailed
t, df	t=0.8421 df=2	t, df	t=3.583 df=2	t, df	t=0.4758 df=2	t, df	t=0.5689 df=2
Number of pairs	3	Number of pairs	3	Number of pairs	3	Number of pairs	3
How big is the difference?		How big is the difference?		How big is the difference?		How big is the difference?	
Mean of differences	18.21	Mean of differences	26.49	Mean of differences	7.503	Mean of differences	-9.308
SD of differences	37.46	SD of differences	12.81	SD of differences	27.31	SD of differences	28.34
SEM of differences	21.63	SEM of differences	7.395	SEM of differences	15.77	SEM of differences	16.36
95% confidence interval	-74.85 to 111.3	95% confidence interval	-5.325 to 58.31	95% confidence interval	-60.35 to 75.36	95% confidence interval	-79.7 to 61.09
R squared (partial eta squared)	0.2618	R squared (partial eta squared)	0.8652	R squared (partial eta squared)	0.1017	R squared (partial eta squared)	0.1393
How effective was the pairing?		How effective was the pairing?		How effective was the pairing?		How effective was the pairing?	
Correlation coefficient (r)	0.02275	Correlation coefficient (r)	0.7619	Correlation coefficient (r)	0.9218	Correlation coefficient (r)	-0.8882
P value (one tailed)	0.4928	P value (one tailed)	0.2243	P value (one tailed)	0.1267	P value (one tailed)	0.152
P value summary	ns	P value summary	ns	P value summary	ns	P value summary	ns
Was the pairing significantly effective?	No	Was the pairing significantly effective?	No	Was the pairing significantly effective?	No	Was the pairing significantly effective?	No

Column H	Id4+Id1 AA 30nM each	Column G	Id4+Id2 AA 30nM each	Column F	Id4+Id3 AA 30nM each
vs.	vs.	vs.	vs.	vs.	vs.
Column A	Scrambled 60nM 48h	Column A	Scrambled 60nM 48h	Column A	Scrambled 60nM 48h
Paired t test		Paired t test		Paired t test	
P value	0.2285	P value	0.487	P value	0.9929
P value summary	ns	P value summary	ns	P value summary	ns
Significantly different (P < 0.05)?	No	Significantly different (P < 0.05)?	No	Significantly different (P < 0.05)?	No
One- or two-tailed P value?	Two-tailed	One- or two-tailed P value?	Two-tailed	One- or two-tailed P value?	Two-tailed
t, df	t=1.715 df=2	t, df	t=0.8452 df=2	t, df	t=0.01006 df=2
Number of pairs	3	Number of pairs	3	Number of pairs	3
How big is the difference?		How big is the difference?		How big is the difference?	
Mean of differences	27.77	Mean of differences	20.22	Mean of differences	-0.2859
SD of differences	28.05	SD of differences	41.43	SD of differences	49.25
SEM of differences	16.19	SEM of differences	23.92	SEM of differences	28.43
95% confidence interval	-41.91 to 97.44	95% confidence interval	-82.7 to 123.1	95% confidence interval	-122.6 to 122.1
R squared (partial eta squared)	0.5952	R squared (partial eta squared)	0.2632	R squared (partial eta squared)	0.00005056
How effective was the pairing?		How effective was the pairing?		How effective was the pairing?	
Correlation coefficient (r)	-0.979	Correlation coefficient (r)	-0.9902	Correlation coefficient (r)	-0.5758
P value (one tailed)	0.0653	P value (one tailed)	0.0447	P value (one tailed)	0.3047
P value summary	ns	P value summary	*	P value summary	ns
Was the pairing significantly effective?	No	Was the pairing significantly effective?	Yes	Was the pairing significantly effective?	No

Figure S4G

Column B	Id1 ABC 20nM each	Column N	Id4+Id1 AA 30nM each - Id1	Column D	Id2 ABC 20nM each	Column M	Id4+Id2 AA 30nM each - Id2	Column F	Id3 ABC 20nM each
vs.	vs.	vs.	vs.	vs.	vs.	vs.	vs.	vs.	vs.
Column A	Scrambled 60nM Id1	Column A	Scrambled 60nM Id1	Column C	Scrambled 60nM Id2	Column C	Scrambled 60nM Id2	Column E	Scrambled 60nM Id3
Paired t test		Paired t test		Paired t test		Paired t test		Paired t test	
P value	0.0042	P value	0.0449	P value	0.0005	P value	0.3668	P value	0.059
P value summary	**	P value summary	*	P value summary	***	P value summary	ns	P value summary	ns
Significantly different (P < 0.05)?	Yes	Significantly different (P < 0.05)?	Yes	Significantly different (P < 0.05)?	Yes	Significantly different (P < 0.05)?	No	Significantly different (P < 0.05)?	No
One- or two-tailed P value?	Two-tailed	One- or two-tailed P value?	Two-tailed	One- or two-tailed P value?	Two-tailed	One- or two-tailed P value?	Two-tailed	One- or two-tailed P value?	Two-tailed
t, df	t=15.36 df=2	t, df	t=4.557 df=2	t, df	t=45.66 df=2	t, df	t=1.157 df=2	t, df	t=3.932 df=2
Number of pairs	3	Number of pairs	3	Number of pairs	3	Number of pairs	3	Number of pairs	3
How big is the difference?		How big is the difference?		How big is the difference?		How big is the difference?		How big is the difference?	
Mean of differences	-53.09	Mean of differences	-26.88	Mean of differences	-37.93	Mean of differences	16.76	Mean of differences	-33.95
SD of differences	5.988	SD of differences	10.22	SD of differences	1.439	SD of differences	25.09	SD of differences	14.96
SEM of differences	3.457	SEM of differences	5.899	SEM of differences	0.8306	SEM of differences	14.49	SEM of differences	8.635
95% confidence interval	-67.96 to -38.21	95% confidence interval	-52.26 to -1.5	95% confidence interval	-41.5 to -34.35	95% confidence interval	-45.57 to 79.09	95% confidence interval	-71.11 to 3.203
R squared (partial eta squared)	0.9916	R squared (partial eta squared)	0.9122	R squared (partial eta squared)	0.999	R squared (partial eta squared)	0.401	R squared (partial eta squared)	0.8854
How effective was the pairing?		How effective was the pairing?		How effective was the pairing?		How effective was the pairing?		How effective was the pairing?	
Correlation coefficient (r)	-0.9589	Correlation coefficient (r)	-0.9948	Correlation coefficient (r)	0.9927	Correlation coefficient (r)	-0.7336	Correlation coefficient (r)	0.9999
P value (one tailed)	0.0916	P value (one tailed)	0.0325	P value (one tailed)	0.0384	P value (one tailed)	0.2378	P value (one tailed)	0.0049
P value summary	ns	P value summary	*	P value summary	*	P value summary	ns	P value summary	**
Was the pairing significantly effective?	No	Was the pairing significantly effective?	Yes	Was the pairing significantly effective?	Yes	Was the pairing significantly effective?	No	Was the pairing significantly effective?	Yes

Column L	Id4+Id3 AA 30nM each - Id3	Column H	Id4 ABC 20nM each	Column K	Id4+Id1 AA 30nM each - Id4	Column J	Id4+Id2 AA 30nM each - Id4	Column I	Id4+Id3 AA 30nM each - Id4
vs.	vs.	vs.	vs.	vs.	vs.	vs.	vs.	vs.	vs.
Column E	Scrambled 60nM Id3	Column G	Scrambled 60nM Id4	Column G	Scrambled 60nM Id4	Column G	Scrambled 60nM Id4	Column G	Scrambled 60nM Id4
Paired t test		Paired t test		Paired t test		Paired t test		Paired t test	
P value	0.0583	P value	0.0051	P value	0.0051	P value	0.0241	P value	0.0321
P value summary	ns	P value summary	**	P value summary	**	P value summary	*	P value summary	*
Significantly different (P < 0.05)?	No	Significantly different (P < 0.05)?	Yes	Significantly different (P < 0.05)?	Yes	Significantly different (P < 0.05)?	Yes	Significantly different (P < 0.05)?	Yes
One- or two-tailed P value?	Two-tailed	One- or two-tailed P value?	Two-tailed	One- or two-tailed P value?	Two-tailed	One- or two-tailed P value?	Two-tailed	One- or two-tailed P value?	Two-tailed
t, df	t=3.957 df=2	t, df	t=14.01 df=2	t, df	t=14.01 df=2	t, df	t=6.322 df=2	t, df	t=5.45 df=2
Number of pairs	3	Number of pairs	3	Number of pairs	3	Number of pairs	3	Number of pairs	3
How big is the difference?		How big is the difference?		How big is the difference?		How big is the difference?		How big is the difference?	
Mean of differences	26.52	Mean of differences	-35.87	Mean of differences	-30.01	Mean of differences	-27.91	Mean of differences	-26.74
SD of differences	11.61	SD of differences	4.433	SD of differences	3.71	SD of differences	7.648	SD of differences	8.5
SEM of differences	6.704	SEM of differences	2.56	SEM of differences	2.142	SEM of differences	4.415	SEM of differences	4.907
95% confidence interval	-2.32 to 55.37	95% confidence interval	-46.88 to -24.85	95% confidence interval	-39.23 to -20.8	95% confidence interval	-46.91 to -8.915	95% confidence interval	-47.86 to -5.628
R squared (partial eta squared)	0.8867	R squared (partial eta squared)	0.9899	R squared (partial eta squared)	0.9899	R squared (partial eta squared)	0.9523	R squared (partial eta squared)	0.9369
How effective was the pairing?		How effective was the pairing?		How effective was the pairing?		How effective was the pairing?		How effective was the pairing?	
Correlation coefficient (r)	0.8777	Correlation coefficient (r)	0.9354	Correlation coefficient (r)	0.9841	Correlation coefficient (r)	0.3398	Correlation coefficient (r)	-0.9356
P value (one tailed)	0.1591	P value (one tailed)	0.1151	P value (one tailed)	0.0568	P value (one tailed)	0.3897	P value (one tailed)	0.1149
P value summary	ns	P value summary	ns	P value summary	ns	P value summary	ns	P value summary	ns
Was the pairing significantly effective?	No	Was the pairing significantly effective?	No	Was the pairing significantly effective?	No	Was the pairing significantly effective?	No	Was the pairing significantly effective?	No

Figure S4H

Column F	Id1 ABC 20nM each	Column I	Id4+Id1 AA 30nM each (ID1)	Column E	Id2 ABC 20nM each	Column H	Id4+Id2 AA 30nM each (ID2)
vs.	vs.	vs.	vs.	vs.	vs.	vs.	vs.
Column A	Scrambled 60nM	Column A	Scrambled 60nM	Column A	Scrambled 60nM	Column A	Scrambled 60nM
Unpaired t test		Unpaired t test		Unpaired t test		Unpaired t test	

P value	0.0004	P value	<0.0001	P value	<0.0001	P value	0.7977
P value summary	***	P value summary	****	P value summary	****	P value summary	ns
Significantly different (P < 0.05)?	Yes	Significantly different (P < 0.05)?	Yes	Significantly different (P < 0.05)?	Yes	Significantly different (P < 0.05)?	No
One- or two-tailed P value?	Two-tailed	One- or two-tailed P value?	Two-tailed	One- or two-tailed P value?	Two-tailed	One- or two-tailed P value?	Two-tailed
t, df	t=10.63 df=4	t, df	t=27.33 df=4	t, df	t=21.95 df=4	t, df	t=0.2739 df=4
How big is the difference?		How big is the difference?		How big is the difference?		How big is the difference?	
Mean ± SEM of column A	1.005 ± 0.003384, n=3	Mean ± SEM of column A	1.005 ± 0.003384, n=3	Mean ± SEM of column A	1.004 ± 0.00389, n=3	Mean ± SEM of column A	1.004 ± 0.00389, n=3
Mean ± SEM of column F	0.4218 ± 0.0547, n=3	Mean ± SEM of column I	0.5289 ± 0.01707, n=3	Mean ± SEM of column E	0.2388 ± 0.03464, n=3	Mean ± SEM of column H	1.036 ± 0.1188, n=3
Difference between means	-0.5828 ± 0.05481	Difference between means	-0.4756 ± 0.0174	Difference between means	-0.7651 ± 0.03486	Difference between means	0.03257 ± 0.1189
95% confidence interval	-0.7349 to -0.4306	95% confidence interval	-0.5239 to -0.4273	95% confidence interval	-0.8619 to -0.6683	95% confidence interval	-0.2976 to 0.3627
R squared (eta squared)	0.9658	R squared (eta squared)	0.9947	R squared (eta squared)	0.9918	R squared (eta squared)	0.01841
F test to compare variances		F test to compare variances		F test to compare variances		F test to compare variances	
F, DFn, Dfd	261.3, 2, 2	F, DFn, Dfd	25.45, 2, 2	F, DFn, Dfd	79.27, 2, 2	F, DFn, Dfd	933.2, 2, 2
P value	0.0076	P value	0.0756	P value	0.0249	P value	0.0021
P value summary	**	P value summary	ns	P value summary	*	P value summary	**
Significantly different (P < 0.05)?	Yes	Significantly different (P < 0.05)?	No	Significantly different (P < 0.05)?	Yes	Significantly different (P < 0.05)?	Yes

Column D	Id3 ABC 20nM each	Column G	Id4+Id3 AA 30nM each (ID3)	Column C	Id4 ABC 20nM each	Column G	Id4+Id3 AA 30nM each (ID4)
vs.	vs.	vs.	vs.	vs.	vs.	vs.	vs.
Column A	Scrambled 60nM	Column A	Scrambled 60nM	Column A	Scrambled 60nM	Column A	Scrambled 60nM
Unpaired t test		Unpaired t test		Unpaired t test		Unpaired t test	
P value	<0.0001	P value	0.0009	P value	0.0011	P value	0.0004
P value summary	****	P value summary	***	P value summary	**	P value summary	***
Significantly different (P < 0.05)?	Yes	Significantly different (P < 0.05)?	Yes	Significantly different (P < 0.05)?	Yes	Significantly different (P < 0.05)?	Yes
One- or two-tailed P value?	Two-tailed	One- or two-tailed P value?	Two-tailed	One- or two-tailed P value?	Two-tailed	One- or two-tailed P value?	Two-tailed
t, df	t=33.36 df=4	t, df	t=8.733 df=4	t, df	t=8.484 df=4	t, df	t=10.91 df=4
How big is the difference?		How big is the difference?		How big is the difference?		How big is the difference?	
Mean ± SEM of column A	1.001 ± 0.0002326, n=3	Mean ± SEM of column A	1.001 ± 0.0002326, n=3	Mean ± SEM of column A	1.002 ± 0.0007659, n=3	Mean ± SEM of column A	1.002 ± 0.0007659, n=3
Mean ± SEM of column D	0.3329 ± 0.02002, n=3	Mean ± SEM of column G	0.5872 ± 0.04737, n=3	Mean ± SEM of column C	0.4765 ± 0.0619, n=3	Mean ± SEM of column G	0.5229 ± 0.04388, n=3
Difference between means	-0.668 ± 0.02002	Difference between means	-0.4136 ± 0.04737	Difference between means	-0.5252 ± 0.0619	Difference between means	-0.4788 ± 0.04389
95% confidence interval	-0.7236 to -0.6124	95% confidence interval	-0.5452 to -0.2821	95% confidence interval	-0.6971 to -0.3533	95% confidence interval	-0.6006 to -0.3569
R squared (eta squared)	0.9964	R squared (eta squared)	0.9502	R squared (eta squared)	0.9474	R squared (eta squared)	0.9675
F test to compare variances		F test to compare variances		F test to compare variances		F test to compare variances	
F, DFn, Dfd	7410, 2, 2	F, DFn, Dfd	41473, 2, 2	F, DFn, Dfd	6531, 2, 2	F, DFn, Dfd	3282, 2, 2
P value	0.0003	P value	<0.0001	P value	0.0003	P value	0.0006
P value summary	***	P value summary	****	P value summary	***	P value summary	***
Significantly different (P < 0.05)?	Yes	Significantly different (P < 0.05)?	Yes	Significantly different (P < 0.05)?	Yes	Significantly different (P < 0.05)?	Yes

Column H	Id4+Id2 AA 30nM each (ID4)	Column I	Id4+Id1 AA 30nM each (ID4)	Column B	HPRT 60nM
vs.	vs.	vs.	vs.	vs.	vs.
Column A	Scrambled 60nM	Column A	Scrambled 60nM	Column A	Scrambled 60nM
Unpaired t test		Unpaired t test		Unpaired t test	
P value	0.0039	P value	0.0004	P value	<0.0001
P value summary	**	P value summary	***	P value summary	****
Significantly different (P < 0.05)?	Yes	Significantly different (P < 0.05)?	Yes	Significantly different (P < 0.05)?	Yes
One- or two-tailed P value?	Two-tailed	One- or two-tailed P value?	Two-tailed	One- or two-tailed P value?	Two-tailed
t, df	t=5.996 df=4	t, df	t=10.77 df=4	t, df	t=42.79 df=4
How big is the difference?		How big is the difference?		How big is the difference?	
Mean ± SEM of column A	1.002 ± 0.0007659, n=3	Mean ± SEM of column A	1.002 ± 0.0007659, n=3	Mean ± SEM of column A	1.007 ± 0.003698, n=3
Mean ± SEM of column H	0.475 ± 0.08783, n=3	Mean ± SEM of column I	0.5324 ± 0.04359, n=3	Mean ± SEM of column B	0.1843 ± 0.01886, n=3
Difference between means	-0.5267 ± 0.08784	Difference between means	-0.4693 ± 0.0436	Difference between means	-0.8224 ± 0.01922
95% confidence interval	-0.7706 to -0.2828	95% confidence interval	-0.5904 to -0.3483	95% confidence interval	-0.8758 to -0.7691
R squared (eta squared)	0.8999	R squared (eta squared)	0.9666	R squared (eta squared)	0.9978
F test to compare variances		F test to compare variances		F test to compare variances	
F, DFn, Dfd	13150, 2, 2	F, DFn, Dfd	3239, 2, 2	F, DFn, Dfd	26.01, 2, 2
P value	0.0002	P value	0.0006	P value	0.074
P value summary	***	P value summary	***	P value summary	ns
Significantly different (P < 0.05)?	Yes	Significantly different (P < 0.05)?	Yes	Significantly different (P < 0.05)?	No

Figure S4I

Unpaired t test		
P value		0.000898847
P value summary	***	
Significantly different (P < 0.05)?	Yes	
One- or two-tailed P value?	Two-tailed	
t, df	t=5.128 df=8	
How big is the difference?		
Mean ± SEM of column G	14.12 ± 2.674, n=5	
Mean ± SEM of column H	0.3125 ± 0.3125, n=5	
Difference between means	-13.81 ± 2.693	
95% confidence interval	-20.02 to -7.597	
R squared (eta squared)		0.7667
F test to compare variances		
F, DFn, Dfd	73.25, 4, 4	
P value		0.0011
P value summary	**	
Significantly different (P < 0.05)?	Yes	

Figure S4J

Unpaired t test		
P value		0.0132
P value summary	*	
Significantly different (P < 0.05)?	Yes	
One- or two-tailed P value?	Two-tailed	
t, df	t=3.171 df=8	
How big is the difference?		
Mean ± SEM of column J	50 ± 13.94, n=5	
Mean ± SEM of column M	4 ± 4, n=5	
Difference between means	-46 ± 14.51	
95% confidence interval	-79.45 to -12.55	
R squared (eta squared)		0.5569
F test to compare variances		
F, DFn, Dfd	12.15, 4, 4	
P value		0.0329
P value summary	*	
Significantly different (P < 0.05)?	Yes	

Figure S5A

	Counts per Million					
**Gene name**	**BLO567A1_FGF_GFP_1**	**BLO567A2_FGF_GFP_2**	**BLO567A3_FGF_GFP_3**	**BLO567A10_FGF_ID4_1**	**BLO567A11_FGF_ID4_2**	**BLO567A12_FGF_ID4_3**
Ascl1	1730	1392	1516	1802	1403	939
Tcf3	3333	3076	2916	2152	2018	2362
Tcf4	22871	25329	24487	28622	27349	29468
Tcf12	9981	13518	10662	7227	9800	8127
**Hes1**	1253	700	878	954	813	1415
**Hes5**	101	16	26	10	11	8
**Hey1**	784	297	658	598	395	420

Figure S6C

Unpaired t test		
P value		0.0075
P value summary	**	
Significantly different (P < 0.05)?	Yes	
One- or two-tailed P value?	Two-tailed	
t, df	t=2.709 df=161	
How big is the difference?		
Mean ± SEM of column A	6.425 ± 0.5706, n=80	
Mean ± SEM of column B	4.422 ± 0.4739, n=83	
Difference between means	-2.003 ± 0.7394	
95% confidence interval	-3.464 to -0.5431	
R squared (eta squared)		0.0436
F test to compare variances		
F, DFn, Dfd	1.397, 79, 82	
P value		0.1347
P value summary	ns	
Significantly different (P < 0.05)?	No	

Figure S6E

Unpaired t test		
P value		0.2373
P value summary	ns	
Significantly different (P < 0.05)?	No	
One- or two-tailed P value?	Two-tailed	
t, df	t=1.668 df=2	
How big is the difference?		
Mean ± SEM of column A	2.812 ± 1.982, n=2	
Mean ± SEM of column B	6.379 ± 0.8031, n=2	
Difference between means	3.567 ± 2.139	
95% confidence interval	-5.635 to 12.77	
R squared (eta squared)		0.5817
F test to compare variances		
F, DFn, Dfd		
P value		
P value summary		
Significantly different (P < 0.05)?		

Figure S6F

Unpaired t test		
P value		0.3493
P value summary	ns	
Significantly different (P < 0.05)?	No	
One- or two-tailed P value?	Two-tailed	
t, df	t=1.212 df=2	
How big is the difference?		
Mean ± SEM of column E	34.14 ± 14.14, n=2	
Mean ± SEM of column F	54.43 ± 8.98, n=2	
Difference between means	20.3 ± 16.75	
95% confidence interval	-51.77 to 92.36	
R squared (eta squared)		0.4234
F test to compare variances		
F, DFn, Dfd		
P value		
P value summary		
Significantly different (P < 0.05)?		

Figure S6G

P65 RGLs/mm3

Tukey's multiple comparisons test	Mean Diff.	95.00% CI of diff.	Significant?	Summary
Id4 +/fl vs. Id4 fl/fl	4139	-4717 to 12996	No	ns
Id4 +/fl vs. Combined Id4wt	2452	-6884 to 11787	No	ns
Id4 fl/fl vs. Combined Id4wt	-1688	-10544 to 7169	No	ns

Test details	Mean 1	Mean 2	Mean Diff.	SE of diff.
Id4 +/fl vs. Id4 fl/fl	32120	27981	4139	2779
Id4 +/fl vs. Combined Id4wt	32120	29669	2452	2929
Id4 fl/fl vs. Combined Id4wt	27981	29669	-1688	2779

Adjusted P Value	
0.5872	B-C
0.9138	B-E
0.9712	C-E

n1	n2	q	DF
4	5	2.107	12
4	4	1.184	12
5	4	0.8589	12

P90 RGLs/mm3

Tukey's multiple comparisons test	Mean Diff.	95.00% CI of diff.	Significant?	Summary
Id4 +/fl vs. Id4 fl/fl	-3799	-11323 to 3726	No	ns
Id4 +/fl vs. combined Id4 wt	-2261	-9786 to 5263	No	ns
Id4 fl/fl vs. combined Id4 wt	1537	-6322 to 9397	No	ns

Test details	Mean 1	Mean 2	Mean Diff.	SE of diff.
Id4 +/fl vs. Id4 fl/fl	23116	26915	-3799	2456
Id4 +/fl vs. combined Id4 wt	23116	25377	-2261	2456
Id4 fl/fl vs. combined Id4 wt	26915	25377	1537	2565

Adjusted P Value	
0.5493	B-C
0.8848	B-E
0.9732	C-E

n1	n2	q	DF
6	5	2.187	16
6	5	1.302	16
5	5	0.8476	16

Figure S6H

Unpaired t test		
P value		0.0072
P value summary	**	
Significantly different (P < 0.05)?	Yes	
One- or two-tailed P value?	Two-tailed	
t, df	t=5.054 df=4	
How big is the difference?		
Mean ± SEM of column A	47.48 ± 7.392, n=3	
Mean ± SEM of column B	88.02 ± 3.117, n=3	
Difference between means	40.55 ± 8.022	
95% confidence interval	18.27 to 62.82	

Figure S6I

Unpaired t test	
P value	<0.0001
P value summary	****
Significantly different (P < 0.05)?	Yes
One- or two-tailed P value?	Two-tailed
t, df	t=17.27 df=4
How big is the difference?	
Mean ± SEM of column A	11.24 ± 2.941, n=3
Mean ± SEM of column D	74.4 ± 2.173, n=3
Difference between means	63.16 ± 3.657
95% confidence interval	53.01 to 73.31

R squared (eta squared)		0.8646	R squared (eta squared)		0.9868
F test to compare variances			F test to compare variances		
F, DFn, Dfd	5.622, 2, 2		F, DFn, Dfd	1.833, 2, 2	
P value		0.302	P value		0.7061
P value summary	ns		P value summary	ns	
Significantly different (P < 0.05)?	No		Significantly different (P < 0.05)?	No	
