# Supplementary material for: Id4 promotes the elimination of the pro-activation factor Ascl1 to maintain quiescence of adult hippocampal stem cells
Source: eLife. 2019 Sep 25;8:e48561. doi: 10.7554/eLife.48561 (PMC6805120; doi:10.7554/eLife.48561)
Supplement: Supplementary file 1. [file elife-48561-supp1.docx]

# Supplementary File 1. Key resources table

| **Reagent type (species) or resource** | **Designation** | **Source or reference** | **Identifiers** | **Additional information** |
| --- | --- | --- | --- | --- |
| Antibody | Rabbit polyclonal anti-Actin | Sigma-Aldrich | ID_source:A2066; ID_source:RRID:AB_476693 | WB(1:1000) |
| Antibody | Mouse monoclonal anti-Ascl1 | BD Pharmingen | ID_source:556604; ID_source:RRID:AB_396479 | WB(1:500), ICC(1:100) |
| Antibody | Rabbit monoclonal anti-Ccnd1 | ThermoScientific | ID_source:RM-9104; ID_source:RRID:AB_720758 | ICC(1:400) |
| Antibody | Mouse monoclonal anti-E47 ((E2A (Yae)) | Santa Cruz Biotechnology | ID_source:sc-416; ID_source:RRID:AB_627472 | WB(1:200), IP(1:225) |
| Antibody | Rat monoclonal anti-DYKDDDDK tag epitope (anti-FLAG) | BioLegend | ID_source:637301, Clone L5; ID_source:RRID:AB_1134266 | ICC(1:200) |
| Antibody | Rat monoclonal anti-GFAP | Invitrogen | ID_source:Cat# 13-0300; ID_source:RRID:AB_86543 | ICC(1:500) |
| Antibody | Chicken polyclonal anti-GFP | Abcam | ID_source:ab13970; ID_source:RRID:AB_300798 | ICC(1:2000) |
| Antibody | Rabbit polyclonal anti-GFP | Life Technologies | ID_source:A11122; ID_source:RRID:AB_221569 | WB(1:1000), IP(1:1000) |
| Antibody | Rabbit polyclonal anti-HA tag | Abcam | ID_source:ab9110; ID_source:RRID:AB_307019 | IP(1:1000) |
| Antibody | Rabbit monoclonal anti-Id1 | Biocheck | ID_source:BCH-1/#37-2; ID_source:RRID:AB_2713996 | WB(1:500), ICC(1:1000) |
| Antibody | Rabbit monoclonal anti-Id2 | Biocheck | ID_source:BCH-3/#9-2-8 | ICC(1:1000) |
| Antibody | Rabbit monoclonal anti-Id3 | Biocheck | ID_source:BCH-4/#17-3 | WB(1:500), ICC(1:200) |
| Antibody | Rabbit monoclonal anti-Id4 | Biocheck | ID_source:BCH-9/#82-12 | WB(1:2500), IP(1:400), ICC(1:1000) |
| Antibody | Mouse monoclonal anti-Ki67 | BD Biosciences | ID_source:550609 | ICC(1:50) |
| Antibody | Rabbit monoclonal anti-Ki67 | ThermoScientific | ID_source:RM-9106-R7; ID_source:RRID:AB_149920 | ICC(1:200) |
| Antibody | Mouse monoclonal anti-Nestin | Millipore | ID_source:Mab353; ID_source:RRID:AB_94911 | ICC(1:100) |
| Antibody | Rat monoclonal anti-Sox2 | Invitrogen | ID_source:14-9811-82; ID_source:RRID:AB_11219471 | ICC(1:400) |
| Antibody | Rabbit monoclonal anti-Tcf4 | Abcam | ID_source:NCI-R159-6 / ab217668; ID_source:RRID:AB_2714172 | ICC(1:2000) |
| Antibody | Goat polyclonal anti-tdTomato | Sicgen | ID_source:AB8181-200; ID_source:RRID:AB_2722750 | ICC(1:1000) |
| Antibody | Rabbit polyclonal anti-Tuj1 | BioLegend (previously Covance) | ID_source:PRB-435P-100; ID_source:RRID:AB_291637 | ICC(1:400) |
| Antibody | Mouse monoclonal anti-V5 | ThermoFisher Scientific | ID_source:R960-25; ID_source:RRID:AB_2556564 | IP(1:1000) |
| Secondary antibody | Alexa Fluor® 488 AffiniPure Donkey Anti-Chicken IgY (IgG) (H+L) | Jackson | ID_source:703-545-155; ID_source:RRID:AB_2340375 | (1:500) |
| Secondary antibody | Alexa Fluor® 488 AffiniPure F(ab')₂ Fragment Donkey Anti-Mouse IgG (H+L) | Jackson | ID_source:715-546-151; ID_source:RRID:AB_2340850 | (1:500) |
| Secondary antibody | Alexa Fluor® 488 AffiniPure F(ab')₂ Fragment Donkey Anti-Rat IgG (H+L) | Jackson | ID_source:712-546-150; ID_source:RRID:AB_2340685 | (1:500) |
| Secondary antibody | Cy™3 AffiniPure F(ab')₂ Fragment Donkey Anti-Rabbit IgG (H+L) | Jackson | ID_source:711-166-152; ID_source:RRID:AB_2313568 | (1:500) |
| Secondary antibody | Cy™3 AffiniPure F(ab')₂ Fragment Donkey Anti-Mouse IgG (H+L) | Jackson | ID_source:715-166-151; ID_source:RRID:AB_2340817 | (1:500) |
| Secondary antibody | Alexa Fluor® 647 AffiniPure F(ab')₂ Fragment Donkey Anti-Rat IgG (H+L) | Jackson | ID_source:712-606-153; ID_source:RRID:AB_2340696 | (1:500) |
| Secondary antibody | Alexa Fluor® 647 AffiniPure Donkey Anti-Goat IgG (H+L) | Jackson | ID_source:705-605-147; ID_source:RRID:AB_2340437 | (1:500) |
| Secondary antibody | Alexa Fluor® 647 AffiniPure F(ab')₂ Fragment Donkey Anti-Mouse IgG (H+L) | Jackson | ID_source:715-606-151; ID_source:RRID:AB_2340866 | (1:500) |
| Antibody | Polyclonal Rabbit Anti-Mouse Immunoglobulins/HRP | Dako | ID_source:P0161; ID_source:RRID:AB_2687969 | (1:1000) |
| Antibody | Polyclonal Goat Anti-Rabbit Immunoglobulins/HRP | Dako | ID_source:P0448; ID_source:RRID:AB_2617138 | (1:1000) |
| Sequence-based reagent | RNAscope® Probe Mm-Ascl1-C2 | ACD (Bio-Techne) | ID_source:313291-C2 | (1:50) in probe diluent or C1-probe |
| Sequence-based reagent | RNAscope® Probe Mm-Mki67-C1 | ACD (Bio-Techne) | ID_source:416771 | (1:1) |
| Sequence-based reagent | RNAscope® Probe Mm-Id4-C1 | ACD (Bio-Techne) | ID_source:447861 | (1:1) |
| Commercial assay, kit | TSA® Plus fluorescein | Perkin Elmer | ID_source:PN NEL741001KT |  |
| Commercial assay, kit | TSA® Plus Cyanine 3 | Perkin Elmer | ID_source:PN NEL744001KT |  |
| Commercial assay, kit | RNeasy® Mini Kit | Qiagen | ID_source:74104 |  |
| Commercial assay, kit | Direct-zol™RNA MiniPrep | Zymo Research | ID_source:R2052 |  |
| Commercial assay, kit | High Capacity cDNA Reverse Transcription Kit | Applied Biosystems | ID_source:4387406 |  |
| Commercial assay, kit | Click-iT® EdU Alexa Fluor 647 Imaging Kit | Invitrogen | ID_source:C10340 |  |
| Commercial assay, kit | Amaxa mouse neural stem cell nucleofector kit | Lonza | ID_source:VPG-1004 |  |
| Commercial assay, kit | RNAscope® Multiplex Fluorescent Reagent Kit V2 | ACD Bio-Techne | ID_source:323110 |  |
| Commercial assay, kit | TaqMan™ Gene Expression MasterMix | Applied Biosystems | ID_source:4369016 |  |
| Peptide, recombinant protein | Recombinant Mouse BMP-4 | R&D Systems | ID_source:5020-BP-010 |  |
| Peptide, recombinant protein | FGF2 | Peprotech | ID_source:450-33 |  |
| Peptide, recombinant protein | Laminin | Sigma | ID_source:L2020 |  |
| Peptide, recombinant protein | Heparin | Sigma | ID_source:H3393-50KU |  |
| Other | DMEM/F-12 + Glutamax | Invitrogen | ID_source:31331-093 |  |
| Other | N2 supplement | R&D Systems | ID_source:AR009 |  |
| Other | Lysis Buffer | ThermoFischer Scientific | ID_source:87788 |  |
| Other | Protease inhibitor cocktail | ThermoFischer Scientific | ID_source:87786 |  |
| Other | EDTA | ThermoFischer Scientific | ID_source:87788 |  |
| Other | Phosphatase inhibitor cocktail | ThermoFischer Scientific | ID_source:78420 |  |
| Other | Laemmli sample buffer | Sigma | ID_source:S3401-10VL |  |
| Other | Protein G Sepharose | Sigma | ID_source:P3296 |  |
| Commercial assay, kit | ECL Western Blotting Reagents | Sigma | ID_source:GERPN2106 |  |
| Chemical compound, drug | MG-132(R) | Sigma | ID_source:SML1135-5MG |  |
| Chemical compound, drug | 4-hydroxytamoxifen | Sigma | ID_source:H6278-50MG |  |
| Software, algorithm | Raw and analyzed data | This paper | GEO: GSE116997  Token for reviewers: wpgrgmkgzpcznyx | See Materials and Methods “RNA sequencing and analysis” |
| Cell line (*Mus musculus*), (male) | Primary adult hippocampal WT neural stem cell line #5 | This paper | n/a | Freshly isolated and maintained in F. Guillemot lab; See Materials and Methods “Primary cell cultures” |
| Cell line (*M. musculus*), (male) | Primary adult hippocampal *Ascl1Venus* neural stem cell line | This paper | n/a | Freshly isolated and maintained in F. Guillemot lab; See Materials and Methods “Primary cell cultures” |
| Cell line (*M. musculus*) | Primary adult hippocampal *Ascl1KiGFP* neural stem cell line | This paper | n/a | Freshly isolated and maintained in F. Guillemot lab; See Materials and Methods “Primary cell cultures” |
| Cell line (*M. musculus*), (male) | Primary adult hippocampal *Huwe1flx* (Huwe1^tm1Alas^) neural stem cell line | This paper | n/a | Freshly isolated and maintained in F. Guillemot lab; See Materials and Methods “Primary cell cultures” |
| Strain, strain background (*M. musculus*) | Slc1a3^tm1(cre/ERT2)Mgoe^ (Glast-CreERT2) | Mori et al., 2006 | ID_source:RRID:MGI:5466676 |  |
| Strain, strain background (*M. musculus*) | Gt(ROSA)26Sor^tm1(EYFP)Cos^ (RYFP) | Srinivas et al., 2001 |  |  |
| Strain, strain background (*M. musculus*) | Gt(ROSA)26Sor^tm9(CAG-tdTomato)Hze^ (tdTomato) | Madisen et al., 2010 |  |  |
| Strain, strain background (*M. musculus*) | Ascl1^tg1(venus)Rik^ (Ascl1Venus) | Imayoshi et al., 2015 |  |  |
| Strain, strain background (*M. musculus*) | Ascl1^tm1Reed^ (Ascl1KiGFP) | Leung et al., 2007 |  |  |
| Strain, strain background (*M. musculus*) | Id4 flx | Best et al., 2014 |  |  |
| Strain, strain background (*M. musculus*) | Glast-CreERT2;Id4flx;RYFP | E. Huillard |  |  |
| Strain, strain background (*M. musculus*) | Glast-CreERT2;tdTomato | This paper |  | See Materials and Methods “Mouse models” |
| Strain, strain background (*M. musculus*) | Glast-CreERT2;Id4flx;tdTomato | This paper |  | See Materials and Methods “Mouse models” |
| Sequence-based reagent | ACTB QPCR probe | Applied Biosystems | ID_source:4352933E |  |
| Sequence-based reagent | Mm03058063_m1 Ascl1 | Applied Biosystems | ID_source:4331182 |  |
| Sequence-based reagent | Mm01279269_m1 Dll1 | Applied Biosystems | ID_source:4331182 |  |
| Sequence-based reagent | GAPDH QPCR probe | Applied Biosystems | ID_source:4352932E |  |
| Sequence-based reagent | Mm00775963_g1 Id1 | Applied Biosystems | ID_source:4331182 |  |
| Sequence-based reagent | Mm00711781_m1 Id2 | Applied Biosystems | ID_source:4331182 |  |
| Sequence-based reagent | Mm00492575_m1 Id3 | Applied Biosystems | ID_source:4331182 |  |
| Sequence-based reagent | Mm00499701_m1 Id4 | Applied Biosystems | ID_source:4331182 |  |
| Sequence-based reagent | Id1 (mouse) siRNA | Origene | ID_source:SR403015 |  |
| Sequence-based reagent | Id2 (mouse) siRNA | Origene | ID_source:SR402424 |  |
| Sequence-based reagent | Id3 (mouse) siRNA | Origene | ID_source:SR401838 |  |
| Sequence-based reagent | Id4 (mouse) siRNA | Origene | ID_source:SR508104 |  |
| Sequence-based reagent | Scrambled siRNA | Origene | ID_source:SR508104 |  |
| Sequence-based reagent | HPRT siRNA | Origene | ID_source:SR405844 |  |
| Recombinant DNA reagent | pBABE-N-FLAG-hId4-Puro (plasmid) | Rahme, GJ., Israel, MA., 2015 | n/a |  |
| Recombinant DNA reagent | pBABE-empty-Puro (plasmid) | Rahme, GJ., Israel, MA., 2015 | n/a |  |
| Recombinant DNA reagent | pBABE-N-FLAG-hID1-Puro (plasmid) | Rahme, GJ., Israel, MA., 2015 | n/a |  |
| Recombinant DNA reagent | pBABE-N-FLAG-hID2-Puro (plasmid) | Rahme, GJ., Israel, MA., 2015 | n/a |  |
| Recombinant DNA reagent | pBABE-N-FLAG-hID3-Puro (plasmid) | Rahme, GJ., Israel, MA., 2015 | n/a |  |
| Recombinant DNA reagent | pBABE-N-FLAG-hID4-Puro (plasmid) | Rahme, GJ., Israel, MA., 2015 | n/a |  |
| Recombinant DNA reagent | pcβ-Id4-FLAG (plasmid) | Rahme, GJ., Israel, MA., 2015 | n/a |  |
| Recombinant DNA reagent | pCAGGS-E47-IRES-GFP (plasmid) | This paper | n/a | See Materials and Methods “Cell treatments, constructs and transfection” |
| Recombinant DNA reagent | pCAGGS-IRES-GFP (plasmid) | This paper | n/a | See Materials and Methods “Cell treatments, constructs and transfection” |
| Recombinant DNA reagent | pmaxGFP Vector (Amaxa mouse neural stem cell nucleofector kit) (plasmid) | Lonza | ID_source:VPG-1004 |  |
| Software, algorithm | FIJI v1.0 | Schindelin et al., 2012 | <https://fiji.sc> |  |
| Software, algorithm | GraphPad Prism 7 | GraphPad Software | <https://www.graphpad.com/scientific-software/prism/> |  |
| Software, algorithm | DAVID Bioinformatics Resource v6.8 | Huang et al., 2009 | <https://david.ncifcrf.gov> |  |
| Software, algorithm | FastQC | Andrews., 2010 | <https://www.bioinformatics.babraham.ac.uk>  /projects/fastqc/ |  |
| Software, algorithm | Trimmomatic | Bolger et al., 2014 | <https://github.com/timflutre/trimmomatic> |  |
| Software, algorithm | tophat2 | Kim, D., et al., 2013 | <https://ccb.jhu.edu/software/tophat> |  |
| Software, algorithm | bowtie2 | Langmead et al., 2012 | <http://bowtie-bio.sourceforge.net/bowtie2/index.shtml> |  |
| Software, algorithm | Cufflinks | Trapnell et al., 2010 | <http://cole-trapnell-lab.github.io/cufflinks/> |  |
| Software, algorithm | Cuffdiff | Trapnell et al., 2013 | <http://cole-trapnell-lab.github.io/cufflinks/cuffdiff/> |  |
| Software, algorithm | HTSeq | Anders et al., 2015 | <https://htseq.readthedocs.io/en/release_0.10.0/> |  |
| Software, algorithm | EDASeq | Risso et al., 2011 | <https://bioconductor.org/packages>  /release/bioc/html/EDASeq.html |  |
| Software, algorithm | RUVseq | Risso et al., 2014 | <https://bioconductor.org/packages>  /release/bioc/html/RUVSeq.html |  |
| Software, algorithm | edgeR | Robinson et al., 2010 | <https://bioconductor.org/packages>  /release/bioc/html/edgeR.html |  |
